# Problem-Based Learning Could Tackle the Issue of Insufficient Education and Adherence in People Living With HIV/AIDS

**DOI:** 10.3389/fphar.2019.00901

**Published:** 2019-08-21

**Authors:** Yang Zhang, Guangyong Xu, Jianhua Hou, Peirong Shi, Suhua Chang, Amos Wu, Aixin Song, Meixia Gao, Xiangpu Cheng, Dan Cui, Hao Wu, Xiaojie Huang, Jie Shi

**Affiliations:** ^1^Center for Infectious Diseases, Beijing You’an Hospital, Capital Medical University, Beijing, China; ^2^National Institute on Drug Dependence, Peking University, Beijing, China; ^3^Dermatological department, Qingdao Infectious Diseases Hospital, Qingdao, China; ^4^Beijing Key Laboratory of AIDS Research, Beijing, China; ^5^Institute of Mental Health/Peking University Sixth Hospital and Key Laboratory of Mental Health, Peking University, Beijing, China; ^6^The Aaron Diamond AIDS Research Center, The Rockfeller University, New York, NY, United States; ^7^Education Department, Beijing You’an Hospital, Capital Medical University, Beijing, China; ^8^Beijing Key Laboratory on Drug Dependence Research, Beijing, China

**Keywords:** problem-based learning (PBL), people living with HIV/AIDS, awareness, adherence, education

## Abstract

**Background:** Poor medication adherence is still the main cause of antiretroviral therapy (ART) failure among people living with HIV/AIDS (PLWHA). Effective behavioral interventions are needed to improve HIV awareness and medication adherence.

**Methods:** In this retrospective cohort study, we assessed the effect of problem-based learning (PBL) approaches to HIV-related education and adherence outcomes among PLWHA and a college student sample. In our study, compared with 309 demography-matched control participants using conventional counseling methods (109 PLWHA and 200 college students), 321 subjects (111 PLWHA and 210 college students) chose to learn HIV-related knowledge *via* PBL-integrated methods. Co-primary outcomes were self-administered questionnaire after HIV-related education by all participants and self-reported medication adherence by newly diagnosed PLWHA, measured in terms of the number of missed doses in the past week at each of the seven visits during a 1-year period. Multivariate regression models adjusting different covariates were used to test the robustness of HIV awareness and adherence association. Mediation model was used to investigate the relationship among PBL training, awareness of HIV, and ART adherence.

**Results:** The knowledge scores of participants in the PBL group were higher than those in the controls (*P* = 0.001), especially the subgroup of newly diagnosed PLWHA in the PBL group (*P* = 0.001). The HIV-related health scores of the PBL college students were also higher than those of subjects exposed to conventional education (*P* < 0.001). There was no significant difference between the two by newly diagnosed PLWHA groups in the number of missed doses during the past week at each visit except at the first follow-up visit (*P* = 0.018). The indirect effect of PBL-integrated education on ART adherence at the 2-week visit through HIV awareness had a point estimate of 0.0349 and a 95% bias-corrected bootstrap confidence interval of 0.0061∼0.0874 in newly diagnosed PLWHA.

**Conclusions:** PLWHA and college students using PBL showed improved awareness of HIV and higher levels of recent ART adherence; however, there was no change in long-term ART adherence in newly diagnosed PLWHA.

## Introduction

The mortality and mobility of people living with HIV/AIDS (PLWHA) have decreased tremendously with the advent of combined antiretroviral therapy (ART) (Gulick et al., 1997; Hammer et al., 1997). In recent years, international guidelines have recommended prompt initiation of ART treatment of HIV infection regardless of level of CD4^+^ T-cell counts (Gunthard et al., 2014; National Center for AIDS/STD Control and Prevention China CDC, 2016; World Health Organization, 2016). A recent systematic review and meta-analysis also showed that early initiation of ART reduced mortality and the likelihood of progression to AIDS, with increased odds of immunological recovery and viral suppression at the ninth month (Song et al., 2018). However, many PLWHA often do not fully comprehend the benefits of early initiation of ART and refuse to accept ART. ART-experienced PLWHA may still exhibit poor compliance and adherence (Bezabhe et al., 2016), which are still the main cause of treatment failure (Paterson et al., 2000; Martin et al., 2008) because of cumulative ART-related toxicity (Song et al., 2018). In fact, the damage of HIV per se is more severe than that of ART (Song et al., 2018). Therefore, to prevent poor ART adherence (Ortego et al., 2011), more effective interventions targeting ART-naive PLWHA patients are needed to improve the awareness of ART-related side effects and toxicity.

Furthermore, a randomized controlled trial (RCT) indicated that earlier initiation of ART could reduce the risk of sexual transmission to HIV-negative counterparts (Cohen et al., 2011). Mathematical modeling simulation also suggested that prompt ART initiation may have a dramatic significant impact on reduced sexual and vertical HIV transmission if treatment coverage and adherence were adequate (Granich et al., 2009). Therefore, a higher level of ART adherence results in effective treatment and prevention, and awareness of HIV and ART facilitates such outcomes.

At the end of 2017, 758,610 HIV/AIDS survivors were reported by the Chinese Center for Disease Control and Prevention, in addition to 134,512 newly diagnosed cases of HIV/AIDS infection (more than 95% of those are sexually transmitted) and 30,718 deaths reported in the same year (AIDS and hepatitis C professional group, Society of infectious diseases, Chinese medical association, Chinese center for disease control and prevention, 2018). A previous cross-sectional survey indicated that around 40∼50% of diagnosed PLWHA progressed to AIDS within 1 year because of late diagnosis (Samet et al., 2001). According to the Chinese integrated HIV/AIDS database, nearly a quarter of all newly diagnosed HIV/AIDS cases were already at World Health Organization (WHO) clinical phase IV (Qiu, 2011). Such late diagnoses, in addition to lack of access to timely and accurate HIV testing (Wu et al., 2015; Harper, 2016), are mainly attributed to a lack of awareness of HIV/AIDS among the public, especially among high-risk populations. Therefore, it is necessary to develop effective strategies to improve the understanding of HIV/AIDS infection among key populations.

Problem-based learning (PBL) is a teaching model, first used in 1969 by Borrows, an American professor of neurology at McMaster University Medical College of Canada. PBL breaks the shackles of the traditional pedagogical methods by promoting teaching, inculcating independent learning skills, and convergent thinking abilities, as well as enhancing the capacity for teamwork and innovative thinking (Solomon et al., 2003; Neville, 2009). Several studies demonstrated that PBL was widely used in HIV/AIDS by health providers, medical students (Solomon et al., 2003; Curran et al., 2005), and researchers (Gandhi et al., 2014). However, there have been few applications of PBL in HIV/AIDS clinical settings and public education. Accordingly, we integrated PBL into conventional health education programs for PLWHA and the general populations, and we evaluated the effectiveness of PBL in promoting HIV awareness and ART adherence among PLWHA participants who were ready for ART initiation.

## Methods

### Study Design

This was an open-label retrospective cohort study. The study design contained two training arms: 1) HIV and ART education for PLWHA and 2) HIV-related health education for the general population (**Figure S1**). Study materials and consent forms were developed in accordance with the Declaration of Helsinki, along with ethical norms, guidelines, and HIV-related laws and regulations in China.

### Study Approval

The study was approved by the Qingdao Infectious Disease Hospital Ethics Committee.

### Study Participants

#### Part 1

Participant enrollment commenced on September 14, 2015, and was completed on December 7, 2016. The study was conducted under the China HIV ART management system (hereinafter referred to as China ART system) in Qingdao, China. According to the China HIV Treatment Guidelines, PLWHA required three sessions of adherence counseling before ART initiation. All of the outpatients selected their education voluntarily and not randomly in real-world settings. Seventy-four newly diagnosed PLWHA (ready to undergo ART) and 37 PLWHA undergoing ART from the China ART system selected PBL combined with direct supervision (PBL group: two sessions of conventional counseling and one session of PBL by newly diagnosed PLWHA and one session of PBL education by the PLWHA undergoing ART during treatment visit). We also recruited a demography-matched active control group with only conventional education (76 new PLWHA exposed three times to conventional adherence education and 33 participants exposed once to conventional adherence education during the treatment visit). Individuals were excluded if they 1) failed questionnaire completion, 2) had less than 9-year education attainment, 3) had diagnoses of chronic diseases requiring routine treatment, and 4) lost to follow-up or discharged.

#### Part 2

During the 2015 and 2016 World AIDS Day campaigns, we included 410 students from universities in Qingdao, China. They were voluntarily divided into PBL (n = 210) and control groups (n = 200), respectively. All of the participants showed adequate comprehension of Chinese materials and completed the questionnaire promptly.

### Study Questionnaire and Study Conduct

#### HIV-Related Education

PBL emphasizes the incorporation of actual clinical cases into teaching materials and provides medical information to students step by step. This method starts with the definition of the problem followed by clinical case analysis, problem solving, group discussion, and guidance from the instructor (Neville, 2009). Here, A, B, C, and D reference materials and questionnaires were designed as “A: Understanding HIV (basic knowledge of HIV epidemics, clinical manifestations, and prevention),” “B: Preparation before ART (necessity, superiority, and adherence of ART),” “C: Surveillance of ART adverse events and visit (adverse reactions and adherence of ART, as well as visit considerations),” and “D: Contact us (contact information for detection, treatment facilities, physicians, nurses, and peer education).” The lessons and questionnaires were based on problems relevant to PLWHA and clinical physicians and independently reviewed by three HIV experts. Suggestions from public health physicians, volunteers, and PLWHA were also considered and integrated.

In Part 1, newly diagnosed PLWHA in the PBL group were educated *via* PBL after twice routine adherence education. Under the guidance of physician or nurse (1 physician/nurse: 1 patient), the participants actively learned the tutorials and answered the material-related questions independently. Participants undergoing ART in the PBL group completed PBL education and assessment at the latest visit. At the same time, newly diagnosed PLWHA in the control group were required to complete the same test after three sessions of regular HIV education, and participants undergoing treatment in the control group were exposed to conventional education and answered questionnaires at the latest follow-up. Each patient in the two groups spent about 30∼60 min per session and another 30 min on evaluation (including Q & A).

In Part 2, college students in the PBL group learned A and D sessions [1 physician (teacher): 10 students] and completed the corresponding questionnaire. The control group finished the examination after receiving regular HIV education. The training program lasted about 30 min, and the response to questionnaires (including Q & A) lasted about 30 min in the two groups.

#### Follow-Up Assessments

In Part 1, the follow-up visits for self-reported medication adherence by new participants were evaluated according to the number of missed doses during the past week at each visit (2 weeks, 1 month, 2 months, 3 months, 6 months, 9 months, and 1 year after education) in the China ART system.

### Outcomes

The co-primary outcomes included self-administered questionnaire scores after HIV-related education. The secondary outcome included self-reported medication adherence for newly diagnosed PLWHA, measured by the number of missed doses during the past week at each visit in the China ART system.

### Statistical Analysis

The HIV-related education score and the number of missed doses in the past week of each visit were represented by mean ± standard deviation (SD) and median in each group, respectively. The averaged AIDS questionnaire scores showing normal distribution were validated by Shapiro–Wilk test and compared using *t* test. The number of missed doses in the past week at each visit not conforming to normal distribution was compared by Wilcoxon rank sum test. Linear regression was used for the analysis of intervention, age, gender, CD4^+^ T-cell counts, and AIDS questionnaire scores. Because awareness of HIV and ART adherence are potentially correlated, a series of multivariable linear regression models were adopted to analyze the effectiveness of PBL or HIV awareness on ART adherence after adjustment for potential confounders. In model 1, only the study method (newly diagnosed PLWHA of PBL group compared with control group), age, gender, CD4^+^ T-cell counts, sulfamethoxazole treatment (used to prevent opportunistic infections, increasing the number and frequency of medications), and frequency of daily ART (once for tenofovir or twice for zidovudine per day) were included. Model 2 added the AIDS questionnaire scores to model 1. Model 3 was identical to model 2 except in the study method. Mediation analysis was tested using bootstrapping of model 4 in PROCESS (Hayes, 2013) to analyze the sampling distribution of the indirect effect with a threshold of *P* < 0.05. The HIV education method (with or without PBL) was used as the independent variable (*X*); ART adherence at the 2-week follow-up (the number of missed doses times in the past week) was used as dependent variable (*Y*); and the awareness of HIV (the average AIDS questionnaire score) was used as mediator (*M*). Gender, age, and CD4^+^ T-cell counts were used as covariates. N = 10,000 was used for bootstrap. All of the analyses were performed in SPSS 21.0 statistical software.

## Results

### Sociodemographic Findings

In Part 1, participants had a mean age of 33.76 years (SD: 6.28), and 93.64% were male. Around 92.23% participants identified themselves as men who have sex with men (MSM). All participants were in WHO clinical phase I. In Part 2, students had a mean age of 20.21 years (SD: 2.47), and 47.56% were female. Gender, age, education, and other general parameters in the corresponding groups of Parts 1 and 2 were not statistically different (*P* > 0.05). The baseline general characteristics of each group were listed in **Table 1**.

**Table 1:** Baseline demographics of participants in each group.

	PBL group	Control group	*P*
PLWHA			
Newly diagnosed participants			
Age (year)^a^	33.14 ± 11.12	32.89 ± 8.51	>0.05
Gender (male/female)^b^	70/4	72/4	>0.05
CD4^+^ T-cell counts^a^	389.63 ± 172.16	359.36 ± 155.59	>0.05
Compound sulfamethoxazole (%)	10.81%	2.63%	>0.05
Zidovudine therapy (%)	6.76%	6.58%	>0.05
Participants in treatment			
Age (year)^a^	34.28 ± 10.85	35.42 ± 9.76	>0.05
Gender (male/female)^b^	34/3	30/3	>0.05
CD4^+^ T-cell counts^a^	363.22 ± 166.19	352.17 ± 159.23	>0.05
College students			
Age (year)^a^	20.43 ± 3.02	20.02 ± 2.69	>0.05
Gender (male/female)^b^	99/111	96/104	>0.05

PBL, problem-based learning; PLWHA, people living with HIV/AIDS. ^a^The data showing normal distribution using Shapiro–Wilk test are represented by the average ± standard deviation. ^b^Number of subjects in each item.

### AIDS Questionnaire Score

The total AIDS questionnaire scores of the participants included in Part 1 of the PBL group were higher than those of the controls (*P* = 0.001). This difference was significantly detected in the newly diagnosed PLWHA (*P* = 0.001) but not in participants on treatment (*P* > 0.05). In Part 2, the HIV-related health scores of the general PBL subjects were also higher than those of the control subjects (*P* < 0.001) (**Table 2**).

**Table 2:** Analysis of AIDS questionnaire in each group.

Group	n	Score (mean ± standard deviation)	*P*
PLWHA			
Total participants in PBL group	111	79.28 ± 11.292	0.001
Total participants in control group	109	74.27 ± 10.925	
Newly diagnosed participants in the PBL group	74	80.00 ± 8.674	0.001
Newly diagnosed participants in the control group	76	74.28 ± 11.127	
Participants in treatment in the PBL group	37	77.84 ± 15.293	0.263
Participants in treatment in the control group	33	74.24 ± 10.616	
College students			
PBL group	210	44.62 ± 7.370	<0.001
Conventional education group	200	39.45 ± 6.196	

PLWHA, people living with HIV/AIDS; PBL, problem-based learning. The data showing normal distribution using Shapiro–Wilk test are represented by the mean ± standard deviation.

With adjustment for age and gender, the multivariate regression model showed that PBL was related to the total AIDS questionnaire score in new participants included in Part 1 (*P* > 0.05; **Table 3**).

**Table 3:** Linear regression model of baseline factors correlated with the AIDS questionnaire score.

Factor	Estimate	*t*	*P*
AIDS education method	−0.287	−3.584	<0.001
Age	−0.128	−1.562	0.12
Gender	0.066	0.813	0.418
CD4^+^ T-cell counts	0.038	0.475	0.636

### ART Adherence

In the seven follow-up appointments within 1 year, the median number of missed doses in the past week was 0 in the two groups of newly diagnosed PLWHA in Part 1. Apart from the first follow-up (*P* = 0.018), no significant difference was found in the other six follow-up appointments between the two groups (*P* > 0.05).

Adjusting for age, gender, sulfamethoxazole treatment, and frequency of daily ART medication, the regression analyses showed that PBL was significantly correlated with ART adherence at the 2-week visit after education in model 1 among newly diagnosed PLWHA in Part 1, and AIDS questionnaire score was significantly associated with ART adherence at 2-week follow-up in models 2 and 3. In addition, regression models 2 and 3 showed that AIDS questionnaire score was significantly correlated with ART adherence at 3-month, 9-month, and 1-year follow-up visits (**Table 4** and **Table S1**).

**Table 4:** Linear regression model for baseline factors correlated with ART adherence at 2 weeks of follow-up.

Follow-up	Model 1			Model 2			Model 3		
	Estimate	*t*	*P*	Estimate	*t*	*P*	Estimate	*t*	*P*
AIDS education method	0.183	2.16	0.032	0.129	1.482	0.141	NA	NA	NA
Age	−0.011	−0.125	0.901	−0.036	−0.419	0.676	−0.053	−0.633	0.528
Gender	0.044	0.526	0.6	0.057	0.691	0.491	0.067	0.805	0.422
CD4^+^ T-cell counts	0.025	0.284	0.777	0.038	0.437	0.662	0.021	0.24	0.81
AIDS questionnaire score	NA	NA	NA	−0.2	−2.339	0.021	−0.235	−2.832	0.005
Compound sulfamethoxazole	−0.054	−0.583	0.561	−0.041	−0.448	0.655	−0.065	−0.72	0.473
Number of daily ART medication doses	0.036	0.402	0.689	0.051	0.585	0.56	0.061	0.691	0.491

A series of multivariable linear regression models were used to assess the effect of PBL or awareness of AIDS on ART adherence after adjustment for potential confounders. In model 1, only study sample (PBL group compared with control group), age, gender, CD4^+^ T-cell counts were included. Besides potential factors that include model 1, the AIDS questionnaire score was also included in model 2. Model 3 was identical to model 2 except for the education form. ART, antiretroviral therapy; NA, not applicable.

### Association Among PBL, HIV Awareness, and ART Adherence

We then analyzed the role of HIV awareness as a mediator for the association between PBL and ART adherence. Given that the HIV education method (with or without PBL) was significantly correlated with both HIV awareness (the average AIDS questionnaire score) and ART adherence at the 2-week follow-up (the number of missed doses in the past week), we hypothesized that the PBL indirectly affected ART adherence at the 2-week follow-up depending on the awareness of HIV. Model 4 in PROCESS was used to test the indirect effect of the path from PBL to the average AIDS questionnaire score to ART adherence. The indirect effect of PBL on ART adherence at the 2-week visit through the HIV awareness had a point estimate of 0.0349 and a 95% bias-corrected bootstrap confidence interval of 0.0061∼0.0874 in newly diagnosed PLWHA recruited in Part 1, which suggested a significant mediation effect (estimates were based on 10,000 bootstrap samples). The indirect effect of PBL on ART adherence was evaluated by the number of missed doses in the past week at the 2-week visit through the HIV awareness, which suggested that the mediation effect was different from zero even at the lower bound of the confidence interval. These data showed that PBL contributed to significant ART adherence, in part by enhancing HIV awareness.

## Discussion

To improve the awareness and adherence of PLWHA, we tested the effectiveness of PBL on PLWHA in China. We generalized this PBL-integrated method to HIV health education in the general population. PLWHA and college students showed significantly improved HIV awareness and recent ART adherence in newly diagnosed PLWHA.

Among PLWHA, the benefits of early initiation of ART treatment outweigh drug-related side effects (Hatano et al., 2013). WHO guidelines recommend ART initiation in PLWHA regardless of CD4^+^ T-cell counts (Eholie et al., 2016). Therefore, it is crucial that PLWHA acquire HIV-related knowledge and appreciate the benefits of good adherence. Formal educational interventions may improve adherence-related knowledge and self- management skills (Patel and Davis, 2006). It is the first time that we adopted PBL education for use among newly PLWHA before ART and during visits. Compared with other conventional adherence education, such as lecture-based learning, mobile applications, messaging, and so forth (Paterson et al., 2000; Martin et al., 2008; Bezabhe et al., 2016), PBL-integrated method has several advantages. First, the real-world clinical issues are addressed by PBL (te Winkel et al., 2006; Kassab et al., 2016). Our participants manifested related clinical problems, which were reflected in the sections of “Understanding HIV” and “Preparation before antiretroviral therapy” in our study. Therefore, the concerns about participants’ condition and ART were much deeper than in the general population and other participants. Second, ART failures in classic cases (Papanna et al., 2013; Woodham et al., 2015) or previous clinical studies were caused by poor adherence to the “Surveillance of ART adverse events and visit,” which was a warning sign to participants. Furthermore, the HIV treatment institutions in China are generally regional specialist hospitals in infectious disease rather than community health service centers or primary care centers (National Center for AIDS/STD Control and Prevention China CDC, 2016). PLWHA often fail to seek help immediately when they experience difficulties with ART, which is contrary to WHO-recommended “service delivery” (World Health Organization, 2016). Therefore, participants’ potential issues and questions were encouraged and addressed by timely assistance from experts and “Contact us” questionnaires.

In our study, PBL was more effective than conventional health education in improving PLWHA awareness of their condition and of ART adherence. Timely telephone feedback from PLWHA who participated in PBL-based comprehensive care showed that the majority supported the new method on the grounds of not just “more understanding of the disease” but also “to enhance the confidence of treatment” and “the feeling that doctors are more intimate.” College students in Part 2 also felt more interested in new methods, and a few cases also impressed them (Papanna et al., 2013; Woodham et al., 2015).

One study involving diabetes care showed that PBL was a cost-effective strategy to obtain physician support for clinical practice guidelines (Benjamin et al., 1999). The PBL group exhibited significant changes in physician adherence with American Diabetes Association standards of care. More importantly, those with poorer glycemic control at baseline realized greater benefit (Benjamin et al., 1999). Although we observed a statistically significant improvement in HIV awareness, the magnitude of this change was likely too small to translate into long-term effect. One possible reason was that both groups showed relatively good ART adherence. Most of the PLWHA were younger and had a higher level of education attainment, and both methods of education achieved the goal of adequate adherence. Another PBL educational program for continuous positive airway pressure (CPAP) used in PLWHA with obstructive sleep apnea (OSA) (Brostrom et al., 2013) showed that participants significantly improved their baseline knowledge about OSA and CPAP after 2 weeks and maintained the training benefit in the 6-month follow-up visit. In any case, PBL may facilitate recent ART adherence in part *via* enhanced awareness of HIV, which is also one of the key components (Bezabhe et al., 2016). Furthermore, participants who manifested a better awareness of HIV tended to show higher adherence in long-term follow-up, without depending on frequent ART medication and regardless of HIV severity.

## Implication to Practice

Therefore, how do PLWHA improve their HIV awareness along with adherence to ART to realize the benefits of treatment and prevention beyond ART adherence? Although the PBL questionnaire in this study received high usability scores, it may not meet the specific needs of PLWHA (Ortego et al., 2011). Therefore, one strategy to resolve this issue is to offer additional HIV-targeted PBL teaching materials, such as Mother-to-Child Transmission and elimination of discrimination or stigma. A recent educational intervention based on the theory of PBL indicated that the Barrows Cards method, which uses at least 15 cards specifically designed to teach management of specific issues by participants, significantly improved adherence to immunosuppressive therapy in adolescents diagnosed with blood cancer with reduced readmissions as well as health care costs (Bagnasco et al., 2016). This method may be used for the structured PBL education of HIV and ART adherence in the future. In addition, regular PBL may also be beneficial to maintain participants’ long-term adherence (Benjamin et al., 1999).

## Study Limitations

Several study limitations should also be addressed. First, this was a retrospective study that could not be used to draw causal conclusions, and the relatively small number of PLWHA in Part 1 in WHO clinical phase I from ART system limited statistical power and lowered the external validity of this study. Therefore, the results should be cautiously interpreted, and further rigorous studies with larger samples are needed to test the robustness and generalization of the current results across diverse patient-centered populations. Second, the PBL teaching material and questionnaire did not cover all aspects of HIV-related knowledge. Thus, a more comprehensive version of teaching materials should be developed and adopted to satisfy the needs of PLWHA. In addition, the self-reported ART adherence data based on ART system were not fully reliable because of social desirability. Thus, self-reported and lab-based blood tests should be combined to enable more reliable assessments for adherence. Finally, the study design was not randomized, and all of the participants selected their educational method based on their preference, which may lower the internal validity of this study without balancing covariates. However, from another perspective, the results originated from real-world clinical setting may be more easily applicable to patient management settings. It is of importance to adopt diversified cohorts across different countries and areas to evaluate the effectiveness of PBL using more rigorous study designs.

## Conclusions

To date, this is the first study to increase the awareness of HIV and improve ART adherence *via* a PBL-based comprehensive care model among HIV-infected and general participants. Compared to the regular care group, we found significant improvement in HIV awareness and short-term ART adherence, but no statistically significant differences were detected in long-term ART adherence.

In summary, despite significant improvement in the awareness of HIV and ART adherence, further well-designed studies are needed to validate the maintenance effects of PBL interventions among PLWHA. Clinicians and health providers should integrate PBL training into regular HIV care to improve the quality of life in PLWHA.

## Ethics Statement

This study was carried out in accordance with the recommendations of guidelines and AIDS-related laws and regulations in China, Qingdao Infectious Disease Hospital Ethics Committee. All subjects gave written informed consent in accordance with the Declaration of Helsinki. The protocol was approved by the Qingdao Infectious Disease Hospital Ethics Committee.

## Author Contributions

YZ, GX, XH, and JS had full access to all of the data in the study and are responsible for the integrity of data and the accuracy of data analysis. Study concept and design: YZ, XH, and JS. Acquisition, analysis, and interpretation of data: YZ, GX, JH, PS, MG, and XH. Drafting the manuscript: YZ, JH, and AW. Critical revision of the manuscript for important intellectual content: PS, SC, MG, XC, DC, HW, XH, and JS. Obtained funding: XC, XH, and HW. Study supervision: DC, XH, and JS. All of the authors gave the publishing approval.

## Funding

This work was supported by the Chinese Government under the 13th Five-Year Plan (No. 2017ZX10201101), the Major Project of Beijing Municipal Science and Technology Committee (No. D161100000416003), the National Natural Science Foundation of China (No. 81701984), the Beijing Postdoctoral Research Foundation (No.2019PC-11), and the President Fund of Capital Medical University (No.13JYIVY142).

## Conflict of Interest Statement

The authors declare that the research was conducted in the absence of any commercial or financial relationships that could be construed as a potential conflict of interest.
