# Supplementary material for: Problem-Based Learning Could Tackle the Issue of Insufficient Education and Adherence in People Living With HIV/AIDS
Source: Front Pharmacol. 2019 Aug 21;10:901. doi: 10.3389/fphar.2019.00901 (PMC6716138; doi:10.3389/fphar.2019.00901)
Supplement: Supplementary file 1 [file DataSheet_1.docx]

***Supplementary Material***

**Problem-based learning could tackle the issue of insufficient education and adherence in people living with HIV/AIDS**

Yang Zhang, PhD, MD ^1, 2, 3, 4^, Guangyong Xu, MD^3^, Jianhua Hou, MS^1^, Peirong Shi, MD^3^, Suhua Chang, PhD^5^, Amos Wu, MS^6^, Aixin Song, BS^1, 4^, Meixia Gao, BS^1^, Xiangpu Cheng, MD ^7^, Dan Cui, MD ^7^, Hao Wu, MD^1, 4^, Xiaojie Huang, MD^1,^ *, Jie Shi, PhD, MD^2, 8,^ *

^1^*Center for Infectious Diseases, Beijing You'an Hospital, Capital Medical University, Beijing 100069, China.*

*^2^National Institute on Drug Dependence, Peking University, Beijing 100191, China*

*^3^Qingdao Infectious Diseases Hospital, Qingdao 266000, China*

*^4^Beijing Key Laboratory of AIDS Research, Beijing 100069, China*

*^5^Institute of Mental Health/Peking University Sixth Hospital and Key Laboratory of Mental Health, Peking University, Beijing 100191, China*

*^6^The Aaron Diamond AIDS Research Center, The Rockfeller University, New York, NY, 10016, United States*

^7^*Education Department, Beijing You'an Hospital, Capital Medical University, Beijing 100069, China.*

*^8^Beijing Key Laboratory on Drug Dependence Research, Beijing 100191, China*

**^*^Correspondence:**

Prof. Jie Shi, National Institute on Drug Dependence, Peking University, 38 Xueyuan Road, Haidian District, Beijing 100191, China. Email: [shijie@bjmu.edu.cn](mailto:shijie@bjmu.edu.cn)

or

Dr. Xiaojie Huang, Center for Infectious Diseases, Beijing You'an Hospital, Capital Medical University, 8 Youanmenwaixitoutiao, Fengtai District, Beijing, 100069, China. Email: [huangxiaojie78@126.com](mailto:huangxiaojie78@126.com)

**Figure S1** Flow diagram of this study. Note: PBL: problem-based learning.

**
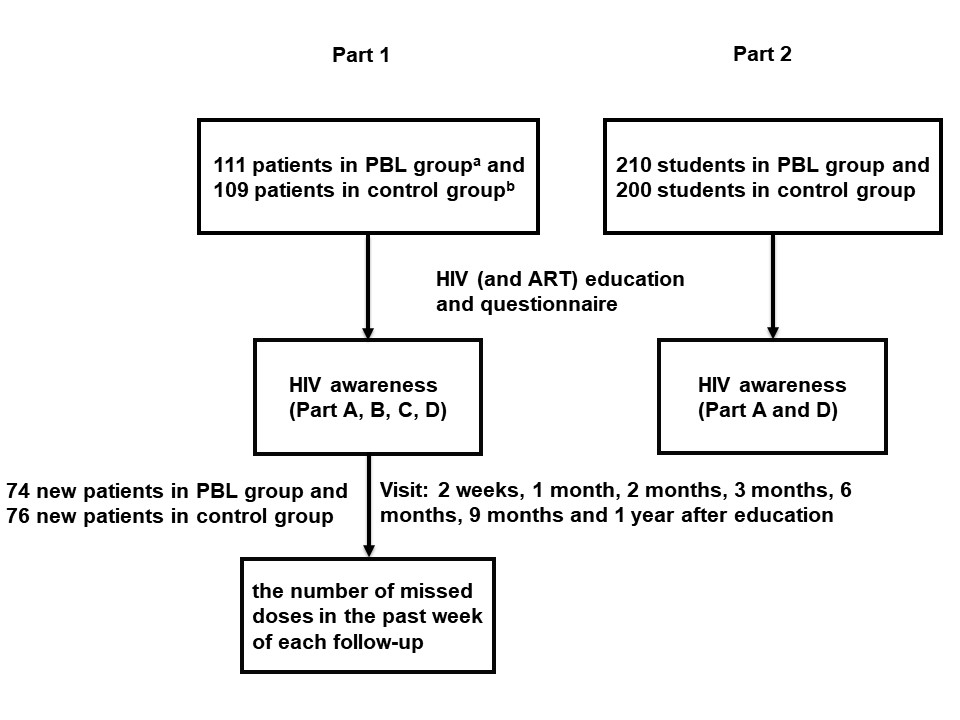
**

**TABLE S1** Linear regression model for baseline factors correlated with ART adherence of other times visit

| Visit | Model 1 | | | Model 2 | | | Model 3 | | |
| --- | --- | --- | --- | --- | --- | --- | --- | --- | --- |
|  | Estimate | *t* | *P* | Estimate | *t* | *P* | Estimate | *t* | *P* |
| 1 month |  |  |  |  |  |  |  |  |  |
| HIV education method | 0.141 | 1.676 | 0.096 | 0.162 | 1.859 | 0.065 | N.A. | N.A. | N.A. |
| Age | 0.054 | 0.642 | 0.522 | 0.064 | 0.751 | 0.454 | 0.042 | 0.489 | 0.626 |
| Gender | -0.069 | -0.833 | 0.406 | -0.074 | -0.892 | 0.374 | -0.062 | -0.744 | 0.458 |
| CD4^+^T cell counts | 0.059 | 0.683 | 0.496 | 0.054 | 0.623 | 0.534 | 0.032 | 0.374 | 0.709 |
| AIDS questionnaire score | N.A. | N.A. | N.A. | 0.079 | 0.915 | 0.362 | 0.036 | 0.426 | 0.671 |
| Compound sulfamethoxazole | -0.029 | -0.318 | 0.751 | -0.035 | -0.373 | 0.71 | -0.065 | -0.709 | 0.48 |
| Times daily medication of ART | 0.205 | 2.337 | 0.021^*^ | 0.199 | 2.259 | 0.025^*^ | 0.211 | 2.379 | 0.019^*^ |
| 2 months |  |  |  |  |  |  |  |  |  |
| HIV education method | 0.12 | 1.404 | 0.162 | 0.093 | 1.051 | 0.295 | N.A. | N.A. | N.A. |
| Age | -0.002 | -0.025 | 0.98 | -0.014 | -0.165 | 0.869 | -0.027 | -0.317 | 0.752 |
| Gender | 0.084 | 0.995 | 0.321 | 0.091 | 1.07 | 0.287 | 0.098 | 1.154 | 0.25 |
| CD4^+^T cell counts | -0.001 | -0.015 | 0.988 | 0.005 | 0.056 | 0.955 | -0.007 | -0.085 | 0.932 |
| AIDS questionnaire score | N.A. | N.A. | N.A. | -0.098 | -1.12 | 0.265 | -0.123 | -1.457 | 0.147 |
| Compound sulfamethoxazole | -0.082 | -0.876 | 0.383 | -0.076 | -0.806 | 0.421 | -0.093 | -1.009 | 0.315 |
| Times daily medication of ART | 0.079 | 0.889 | 0.376 | 0.087 | 0.972 | 0.333 | 0.094 | 1.05 | 0.295 |
| 3 months |  |  |  |  |  |  |  |  |  |
| HIV education method | 0.123 | 1.444 | 0.151 | 0.04 | 0.47 | 0.639 | N.A. | N.A. | N.A. |
| Age | 0.116 | 1.353 | 0.178 | 0.078 | 0.943 | 0.347 | 0.073 | 0.888 | 0.376 |
| Gender | -0.079 | -0.943 | 0.347 | -0.059 | -0.734 | 0.464 | -0.056 | -0.702 | 0.484 |
| CD4^+^T cell counts | -0.072 | -0.831 | 0.407 | -0.053 | -0.632 | 0.528 | -0.058 | -0.703 | 0.483 |
| AIDS questionnaire score | N.A. | N.A. | N.A. | -0.303 | -3.627 | < 0.001^*^ | -0.313 | -3.91 | < 0.001^*^ |
| Compound sulfamethoxazole | -0.095 | -1.017 | 0.311 | -0.075 | -0.837 | 0.404 | -0.083 | -0.938 | 0.35 |
| Times daily medication of ART | 0.049 | 0.55 | 0.583 | 0.072 | 0.848 | 0.398 | 0.075 | 0.887 | 0.377 |
| 6 months |  |  |  |  |  |  |  |  |  |
| HIV education method | 0.096 | 1.127 | 0.261 | 0.069 | 0.785 | 0.434 | N.A. | N.A. | N.A. |
| Age | -0.066 | -0.774 | 0.44 | -0.078 | -0.909 | 0.365 | -0.088 | -1.032 | 0.304 |
| Gender | 0.039 | 0.465 | 0.642 | 0.046 | 0.541 | 0.59 | 0.051 | 0.604 | 0.547 |
| CD4^+^T cell counts | -0.17 | -1.952 | 0.053 | -0.163 | -1.878 | 0.062 | -0.172 | -2.004 | 0.047 |
| AIDS questionnaire score | N.A. | N.A. | N.A. | -0.097 | -1.118 | 0.265 | -0.116 | -1.383 | 0.169 |
| Compound sulfamethoxazole | -0.018 | -0.196 | 0.845 | -0.012 | -0.127 | 0.899 | -0.025 | -0.271 | 0.786 |
| Times daily medication of ART | -0.004 | -0.048 | 0.962 | 0.003 | 0.037 | 0.97 | 0.008 | 0.094 | 0.925 |
| 9 months |  |  |  |  |  |  |  |  |  |
| HIV education method | 0.079 | 0.932 | 0.353 | 0.023 | 0.26 | 0.796 | N.A. | N.A. | N.A. |
| Age | 0.119 | 1.39 | 0.167 | 0.093 | 1.098 | 0.274 | 0.09 | 1.076 | 0.284 |
| Gender | -0.063 | -0.753 | 0.453 | -0.05 | -0.6 | 0.55 | -0.048 | -0.583 | 0.561 |
| CD4^+^T cell counts | -0.08 | -0.915 | 0.362 | -0.066 | -0.774 | 0.441 | -0.069 | -0.818 | 0.415 |
| AIDS questionnaire score | N.A. | N.A. | N.A. | -0.207 | -2.422 | 0.017^*^ | -0.213 | -2.597 | 0.01^*^ |
| Compound sulfamethoxazole | -0.112 | -1.198 | 0.233 | -0.098 | -1.069 | 0.287 | -0.103 | -1.136 | 0.258 |
| Times daily medication of ART | 0.105 | 1.179 | 0.24 | 0.121 | 1.38 | 0.17 | 0.123 | 1.407 | 0.161 |
| 1 year |  |  |  |  |  |  |  |  |  |
| HIV education method | 0.139 | 1.622 | 0.107 | 0.027 | 0.331 | 0.741 | N.A. | N.A. | N.A. |
| Age | -0.004 | -0.052 | 0.959 | -0.055 | -0.688 | 0.493 | -0.059 | -0.744 | 0.458 |
| Gender | -0.061 | -0.718 | 0.474 | -0.034 | -0.434 | 0.665 | -0.032 | -0.411 | 0.682 |
| CD4^+^T cell counts | -0.008 | -0.093 | 0.926 | 0.018 | 0.222 | 0.824 | 0.014 | 0.18 | 0.857 |
| AIDS questionnaire score | N.A. | N.A. | N.A. | -0.408 | -5.04 | < 0.001^*^ | -0.415 | -5.343 | < 0.001^*^ |
| Compound sulfamethoxazole | -0.062 | -0.657 | 0.512 | -0.035 | -0.403 | 0.688 | -0.04 | -0.471 | 0.639 |
| Times daily medication of ART | 0.029 | 0.329 | 0.743 | 0.061 | 0.739 | 0.461 | 0.063 | 0.767 | 0.444 |

Note: Note: A series of multivariable linear regression models were used to assess the effect of PBL or awareness of HIV on ART adherence after adjustment for potential confounders. In model 1, only study sample (PBL group compared with control group), age, gender, CD4+T cell counts were included. Besides potential factors included in model 1, the AIDS questionnaire score was also included in model 2. Model 3 was identical to model 2 except in education form. ART: antiretroviral therapy; N.A.: not applicable.
